# Latent profile analysis of emergency department presentation characteristics in patients with acute ischemic stroke

**DOI:** 10.3389/fneur.2026.1758671

**Published:** 2026-07-09

**Authors:** Xiangguang Yin, Hua Xu, Jing Wei, Ying Li, Ying Chen

**Affiliations:** 1 Department of Emergency Medicine, The Affiliated Hospital of Xuzhou Medical University, Xuzhou Medical University, Xuzhou, Jiangsu Province, China; 2 Department of Nursing, The Affiliated Hospital of Xuzhou Medical University, Xuzhou Medical University, Xuzhou, Jiangsu Province, China

**Keywords:** acute ischemic stroke, clinical outcomes, correlation analysis, emergency department presentation, latent profile analysis, MRS score, multivariate regression

## Abstract

**Objective:**

This study aimed to identify potential profile types characterizing emergency department presentations among patients with acute ischemic stroke (AIS), analyze differences in demographic, clinical features, and clinical outcomes across profiles, and explore associations between presentation characteristics and prognosis (modified Rankin Scale, mRS) to inform precision intervention strategies.

**Methods:**

Using convenience sampling, 220 AIS patients admitted to our hospital from January to December 2024 were enrolled. The Latent Profile Analysis (LPA) model was expanded to include eight indicators capturing multidimensional prehospital behavioral constructs: time from onset to symptom recognition, time from symptom recognition to decision-making, time from onset to presentation, and Stroke Knowledge Awareness Score, emergency medical services (EMS) utilization, living alone status, residential area, and hospital transfer pathway. Latent Profile Analysis (LPA) was performed using Mplus 8.0 software incorporating all eight indicators. Demographic characteristics, clinical features, and clinical outcomes were compared across profiles using χ^2^ tests and Kruskal-Wallis H tests. Spearman correlation analysis assessed the strength of associations between presentation indicators and mRS scores. Multivariate ordinal logistic regression was conducted to examine the independent prognostic value of latent profile membership for mRS, adjusting for age, baseline NIHSS score, comorbidity burden, and onset-to-presentation time.

**Results:**

LPA successfully identified four latent profiles of emergency department presentation characteristics among AIS patients, with optimal model fit (AIC = 7354.379, BIC = 7452.794, Entropy = 0.976). Based on the expanded indicator set, these profiles were recharacterized as: rapid ems-activated profile (31.36%, *n* = 69), delayed recognition-self-presentation profile (27.73%, *n* = 61), family involvement-cautious decision-making profile (16.82%, *n* = 37), and multiple barriers-delayed presentation profile (24.09%, *n* = 53). Significant differences existed among the four profiles in age, education level, residence, living alone status, number of comorbidities, onset time, and NIHSS score (*p* < 0.05). Regarding clinical outcomes, the rapid decision-making-emergency transport group exhibited the highest reperfusion therapy rate (63.77%) and the highest favorable prognosis rate (72.46%). while the multiple barriers-delayed presentation group had the lowest reperfusion rate (7.55%) and highest poor prognosis rate (86.79%) (*p* < 0.001). Correlation analysis revealed significant positive correlations between mRS scores and time from onset to presentation (r = 0.440), time from onset to symptom recognition (r = 0.446), and time from symptom recognition to decision-making (r = 0.458; all *p* < 0.001). Stroke knowledge scores showed a significant negative correlation with mRS scores (r = −0.431, *p* < 0.001). Multivariate regression confirmed latent profile membership as an independent prognostic factor. Compared with the Rapid EMS-Activated Profile, the Multiple Barriers-Delayed Presentation Profile was associated with higher odds of poor prognosis (OR = 4.87, 95%CI:2.92–8.13, *p* < 0.001), independent of age, NIHSS, comorbidity burden, and onset-to-presentation time.

**Conclusion:**

Emergency presentation patterns among AIS patients exhibit significant heterogeneity, allowing classification into four latent profiles with distinct behavioral characteristics. Patients across profiles demonstrate marked differences in presentation efficiency, stroke knowledge levels, and clinical outcomes. Latent profile classification independently predicts functional prognosis beyond traditional clinical confounders. Developing stratified intervention strategies based on core profile characteristics can help reduce presentation delays, enhance treatment efficacy, and provide critical guidance for optimizing emergency management of AIS.

## Introduction

Acute ischemic stroke (AIS), the most prevalent subtype of stroke, is characterized by high incidence, high disability rates, and high mortality. It has become a major public health issue threatening global human health and quality of life (1, 2). Clinical practice confirms that timely and effective reperfusion therapy (such as intravenous thrombolysis or mechanical thrombectomy) is crucial for improving the prognosis of AIS patients (3, 4). However, in China, the timing of treatment for AIS patients remains severely challenged: fewer than 22% of patients arrive at the hospital within 3 h of symptom onset, and after a series of screening and evaluation processes, only 1.6% ultimately receive intravenous thrombolysis (IVT) treatment. The core bottleneck lies in the diverse delays occurring during the emergency department visit process (5, 6). Previous studies indicate that intravenous administration of alteplase within 3 to 4.5 h after AIS symptom onset significantly improves clinical outcomes (7, 8).

It is important to note that emergency department visits by AIS patients are not isolated events but rather a continuous process comprising “symptom recognition-decision to seek care-transportation to medical facilities.” Within this process, patient groups exhibit significant heterogeneity in presentation patterns: some patients rapidly recognize symptoms and opt for emergency transport, minimizing treatment delays; however, a substantial number experience significantly prolonged delays due to insufficient disease awareness, family decision-making hesitation, or inappropriate transport choices, thereby missing the optimal window for intervention (9–11). However, existing studies predominantly employ univariate analyses to explore factors influencing treatment delays, overlooking the overall heterogeneity of treatment patterns. This approach struggles to accurately identify behavioral differences across distinct patient groups. Critically, prior research has largely relied on temporal indicators alone (e.g., onset-to-door time) to characterize delays, without systematically incorporating prehospital behavioral constructs such as EMS utilization, living status, geographic accessibility, and transfer pathways. This limits the ability to distinguish between different mechanisms producing similar delays.

Latent Profile Analysis (LPA), as a continuous variable-based clustering method, can identify latent homogeneous subgroups within data through multidimensional indicators, offering new perspectives for analyzing complex clinical characteristics (12, 13). Previous studies have applied LPA in the AIS field, such as delineating latent profiles of patient psychological capital and survivor anxiety states (14, 15). However, no research has yet employed LPA to analyze the treatment behavior profiles of AIS patients using a comprehensive set of indicators that includes both temporal and behavioral/structural determinants, and there remains a lack of systematic analysis examining the interrelationships among “treatment behavior profiles-clinical characteristics-clinical outcomes,” particularly the quantitative association between distinct presentation patterns and patient prognosis (using mRS scores as the core indicator). In clinical practice, patients’ presentation characteristics—such as symptom recognition speed, decision-making efficiency, and knowledge level-directly influence time to presentation and treatment initiation, thereby impacting prognosis. This logical association has yet to be systematically validated in previous studies using LPA results.

Based on this, this study aims to identify latent profile types of emergency department visit characteristics among AIS patients using LPA, expanding the indicator set to include EMS utilization, living alone status, geographic accessibility (urban/rural residence), and hospital transfer pathway alongside traditional temporal and knowledge indicators, systematically describing the core behavioral features of each profile. It further compares differences in demographic characteristics, clinical indicators, and clinical outcomes among patients in different profiles. The study focuses on exploring the strength of association between indicators related to visit characteristics and patient prognosis (mRS score). The core objective of this study is to clarify the heterogeneous distribution patterns of AIS patients’ presentation characteristics, providing robust theoretical support for developing “stratified, targeted” clinical management strategies. Ultimately, this will contribute to improving the overall treatment outcomes for AIS patients.

## Subjects and methods

### Study population

This study employed convenience sampling to select patients diagnosed with AIS who presented to our hospital’s emergency department between January 2024 and December 2024. (1) Met the diagnostic criteria for AIS outlined in the 2023 Chinese Guidelines for the Diagnosis and Treatment of Acute Ischemic Stroke, with confirmed ischemic lesions identified via cranial computed tomography (CT) or magnetic resonance imaging (MRI); (2) Age ≥ 18 years; (3) Clear time from symptom onset to emergency department arrival (verifiable through patient/family recollection or emergency records); (4) Patients were conscious or had family members/witnesses who could accurately provide information about their presentation; (5) Patients provided informed consent and voluntarily participated in the study. Exclusion criteria: (1) Patients with transient ischemic attack (TIA), hemorrhagic stroke, or other cerebrovascular diseases; (2) Patients with severe hepatic or renal failure, malignancy, psychiatric disorders, or cognitive impairment (e.g., Alzheimer’s disease) that prevented cooperation with the study; (3) Missing clinical data or behavioral information related to the presentation; (4) Patients who were comatose at onset and had no family members/witnesses to provide information. Ultimately, 220 AIS patients meeting the criteria were enrolled in this study.

### Data collection

Data were extracted from the hospital’s electronic medical record (EMR) system and the emergency department stroke registry database. The following indicators were collected: this study rigorously selected four clinically meaningful continuous variables as LPA observational metrics: (1) Time from onset to symptom recognition: The duration (in minutes) from symptom onset to the individual or others recognizing stroke symptoms; (2) Time from symptom recognition to decision-making: The duration (in minutes) from symptom recognition to the decision to seek medical care; (3) Time from onset to presentation: Total time from symptom onset to arrival at the hospital emergency department (minutes); (4) Stroke Knowledge Awareness Score: Measured using a self-developed Stroke Knowledge Scale comprising 15 items covering dimensions such as AIS risk factors (e.g., hypertension, hyperlipidemia), recognition of typical symptoms, and emergency measures (e.g., calling emergency services, avoiding self-medication). Scored on a 1–5 point scale (1 = complete lack of awareness, 5 = complete awareness), with a total score range of 15–75 points. Higher scores indicate better mastery of stroke knowledge; (5) EMS utilization: Recorded as yes/no based on whether the patient arrived at the emergency department via ambulance (emergency medical services); (6) Living alone status: Recorded as yes/no based on whether the patient lived alone at the time of stroke onset; (7) Residential area (geographic accessibility): Classified as urban or rural, serving as a proxy for geographic accessibility to stroke-ready centers and emergency medical resources; (8) Hospital transfer pathway: Classified as direct presentation (patients who came directly to our hospital’s emergency department) versus inter-hospital transfer (patients who were initially transported to another hospital and subsequently transferred to our hospital for stroke-specific care).

#### Other analytical indicators include

Demographic characteristics: Age (45–60 years/61–75 years/>75 years), gender, education level (elementary school or below/junior high and high school/college and above), residence (urban/rural), living alone status (yes/no), occupation (mental laborer/physical laborer/unemployed/retired), marital status (married with spouse/unmarried/divorced/widowed). Clinical Characteristics: Number of underlying conditions (0–1/2–3/≥4), primary underlying conditions (hypertension/hyperlipidemia/diabetes/coronary heart disease), onset time (0:00–6:00/6:00–12:00/12:00–18:00/18:00–24:00), NIHSS score at admission (classified as mild 0–4 points, moderate 5–15 points, severe ≥16 points; higher scores indicate more severe neurological deficits).

#### Clinical outcome measures

Reperfusion therapy (intravenous thrombolysis/mechanical thrombectomy), Length of hospital stay (total days from admission to discharge), Prognosis status (assessed using modified Rankin Scale (mRS) scores at discharge; scores 0–2 indicate favorable prognosis, scores 3–6 indicate unfavorable prognosis; higher mRS scores indicate more severe impairment in activities of daily living).

### Latent profile analysis (LPA)

LPA was conducted using Mplus 8.3 statistical software, incorporating only the aforementioned four continuous variables as observed indicators. The fit indices for the latent profile model comprised three components: (1) Information criteria: Akaike information criterion (AIC), Bayesian information criterion (BIC), adjusted Bayesian information criterion (aBIC). Lower values indicate better model fit; (2) Entropy: Ranging from 0 to 1, values >0.8 indicate good classification accuracy (≥90%); (3) Lo–Mendell–Rubin likelihood ratio test (LMR-LRT) and bootstrapped likelihood ratio test (BLRT): If *p* < 0.05, the k-profile model is significantly superior to the k-1-profile model. Construct LPA models sequentially for 1–5 strata. Compare the AIC, BIC, aBIC, Entropy, and LMR-LRT/BLRT test results across models to select the best-fitting model as the final classification basis.

### Statistical analysis

Data analysis was performed using SPSS 27.0 statistical software (SPSS, Inc., Chicago, IL, United States) and GraphPad Prism 9.5.0 software (GraphPad Software Inc., San Diego, CA, United States). The Shapiro–Wilk test was used to assess normality. Quantitative data are expressed as median (interquartile range) [M (P25, P75)]. Intergroup comparisons were performed using the Kruskal-Wallis H test. Categorical data are presented as number (percentage) [*n* (%)]. Intergroup comparisons were conducted using the chi-square test (to compare differences in characteristics across different presentation profiles). Spearman correlation analysis was used to explore associations between clinical characteristics (time from onset to symptom recognition, time from symptom recognition to decision-making, time from onset to presentation, stroke knowledge score) and outcomes (mRS score). Multivariate ordinal logistic regression was performed to examine the independent prognostic value of latent profile membership for discharge mRS, adjusting for key confounders: age, baseline NIHSS score, comorbidity burden, and onset-to-presentation time. The incremental prognostic value of latent profile classification was evaluated by comparing unadjusted and adjusted models. All statistical tests were two-tailed, with *p* < 0.05 indicating statistical significance.

## Results

### General characteristics of study subjects

This study included 220 patients with AIS. Their demographic and clinical characteristics are summarized in Table 1: 127 males (57.73%) and 93 females (42.27%); Age ranged from 45 to 85 years, with a mean age of (66.59 ± 11.95) years. Among them, 68 patients (30.91%) were aged 45–60 years, 90 patients (40.91%) were aged 61–75 years, and 62 patients (28.18%) were over 75 years old; Educational attainment was predominantly junior high school and high school (78 cases, 35.45%) or college and above (86 cases, 39.09%), with 56 cases (25.45%) at elementary school level or below; Residence was predominantly urban (136 cases, 61.82%), with 84 cases (38.18%) in rural areas; 39 cases (17.73%) lived alone, while 181 cases (82.27%) did not live alone; Occupation distribution showed manual laborers as the largest group (97 cases, 44.09%), followed by unemployed/retired individuals (72 cases, 32.73%) and intellectual workers (51 cases, 23.18%); Marital status was predominantly married with a spouse (185 cases, 84.09%), while unmarried/divorced/widowed individuals accounted for 35 cases (15.91%). Regarding EMS utilization, 98 cases (44.55%) used ambulance transport, and 122 cases (55.45%) used self-transport. Regarding hospital transfer pathway, 152 cases (69.09%) presented directly to our hospital, while 68 cases (30.91%) were inter-hospital transfers.

**Table 1:** General characteristics of study subjects.

Feature classification	Specific categories	Number of instances (*n*)	Percentage (%)
Gender	M	127	57.73%
	F	93	42.27%
Age	45–60 year	68	30.91%
	61–75 year	90	40.91%
	>75 year	62	28.18%
Educational attainment	Elementary school and below	56	25.45%
	Junior high school and high school	78	35.45%
	College degree or above	86	39.09%
Place of residence	City	136	61.82%
	Rural	84	38.18%
Living alone	Yes	39	17.73%
	No	181	82.27%
Occupation	Knowledge workers	51	23.18%
	Manual laborer	97	44.09%
	Unemployed/retired	72	32.73%
Marital status	Married with a spouse	185	84.09%
	Unmarried/divorced/widowed	35	15.91%
EMS utilization	Yes (ambulance)	98	44.55%
	No (self-transport)	122	55.45%
Hospital transfer pathway	Direct presentation	152	69.09%
	Inter-hospital transfer	68	30.91%
Number of underlying conditions	0–1 species	68	30.91%
	2–3 species	95	43.18%
	≥4 species	57	25.91%
Primary underlying disease	Hypertension	161	73.18%
	Hyperlipidemia	104	47.27%
	Diabetes	92	41.82%
	Coronary heart disease	73	33.18%
Onset time	0:00–6:00	30	13.64%
	6:00–12:00	71	32.27%
	12:00–18:00	60	27.27%
	18:00–24:00	59	26.82%
NIHSS score (points)	Mild (0–4 points)	86	39.09%
	Moderate (5–15 points)	114	51.82%
	Severe (≥16 points)	20	9.09%

Regarding the number of comorbidities: 0–1 comorbidities in 68 cases (30.91%); 2–3 comorbidities in 95 cases (43.18%); ≥4 conditions (57 cases, 25.91%). Among primary conditions, hypertension was most prevalent (161 cases, 73.18%), followed by hyperlipidemia (104 cases, 47.27%), diabetes (92 cases, 41.82%), and coronary heart disease (73 cases, 33.18%). Onset times were concentrated between 6:00–12:00 (71 cases, 32.27%), 12:00–18:00 (60 cases, 27.27%), and 18:00–24:00 (59 cases, 26.82%), with the lowest incidence occurring between 0:00 and 6:00 (30 cases, 13.64%); The median NIHSS score at admission was 6.00 (range: 3.00–12.75). Among these, mild (0–4 points) was observed in 86 cases (39.09%), moderate (5–15 points) in 114 cases (51.82%), and severe (≥16 points) in 20 cases (9.09%).

### Results of latent profile model fitting

To determine the optimal number of latent profiles characterizing emergency department visits among AIS patients, models with 1 to 5 profiles were sequentially constructed and their fitting indices compared, as shown in Table 2. As the number of segments increased, AIC, BIC, and aBIC gradually decreased. The 4-segment model achieved the lowest values for AIC (7354.379), BIC (7452.794), and aBIC (7360.893), with Entropy reaching 0.976 (>0.9), indicating extremely high classification accuracy. Simultaneously, the LMR-LRT (*p* = 0.013) and BLRT (*p* = 0.014) tests for the four-profile model achieved statistical significance, indicating its significant superiority over the three-profile model. Based on these comprehensive metrics, the four-profile model was ultimately determined as the optimal fit.

**Table 2:** Comparison of fitting indices for different potential profile models.

Number of cross-sections	AIC	BIC	aBIC	Entropy	LMR-LRT (*P*)	BLRT (*P*)
1	8576.251	8613.581	8578.722			
2	7961.588	8026.067	7965.856	0.973	0.000	0.000
3	7479.889	7561.336	7485.280	0.994	0.000	0.000
4	7354.379	7452.794	7360.893	0.976	0.013	0.014
5	6967.941	7083.324	6975.578	0.971	0.103	0.107

AIC: Akaike information criterion; BIC: Bayesian information criterion; aBIC: adjusted Bayesian information criteriona; LMRT: Lo–Mendell–Rubin likelihood ratio test; BLRT: bootstrapped likelihood ratio test.

### Core characteristics and naming of four potential AIS patient profiles

Based on the fitting results of the four-profile model and the performance of each profile across four core indicators—“time from onset to symptom recognition,” “time from symptom recognition to decision-making,” “time from onset to hospital arrival,” “stroke knowledge awareness score,” “EMS utilization,” “living alone status,” “residential area,” and “hospital transfer pathway”—each profile was characterized and named (Table 3): Profile 1: Rapid decision-making—emergency transport type (*n* = 69, 31.36%): Patients in this category rapidly recognize symptoms after onset (short onset-to-recognition time) and make swift decisions to seek medical care (short recognition-to-decision time). EMS utilization is high (92.8%), with predominantly urban residence (75.4%) and low living-alone rate (7.2%). Direct presentation is the norm (94.2%). They predominantly choose ambulance transport, resulting in significantly shorter onset-to-presentation times compared to other types, along with higher stroke knowledge scores. This indicates that these patients and their families possess strong awareness of stroke symptoms and emergency response capabilities, enabling them to take prompt and appropriate actions. The high EMS use and direct presentation pathway minimize prehospital delays. Profile 2: Delayed recognition-self-seeking care type (*n* = 61, 27.73%): Longer time from onset to symptom recognition and decision-making, with low EMS utilization (24.6%); most ultimately choosing to self-transport to the hospital (non-ambulance transport). Rural residence is highest among profiles (47.5%), and inter-hospital transfer rate is moderate (19.7%). This significantly prolongs the time from onset to presentation, accompanied by lower stroke knowledge scores. This reflects low sensitivity to stroke symptoms and lack of emergency knowledge among these patients and their families, leading to delayed optimal treatment timing. The combination of poor symptom recognition and suboptimal transport mode represents a dual barrier. Profile 3: Family involvement-cautious decision-making type (*n* = 37, 16.82%): The time from onset to symptom recognition was moderate. However, family members exercised caution during the decision-making process, leading to prolonged decision-making time. Transportation methods varied, resulting in moderate time from onset to presentation. Stroke knowledge scores were also moderate. Notably, this profile has the highest inter-hospital transfer rate (35.1%), suggesting that initial presentation to non-stroke centers contributes to delays. This indicates family members play a significant role in healthcare decisions, but the decision-making process may be influenced by multiple factors, slowing down the pace. Profile 4: multiple barriers-delayed seeking care type (*n* = 53, 24.09%): this group exhibited the longest durations across all time dimensions-longest time from onset to symptom recognition, longest decision-making time, and extremely long time from onset to seeking care. They also had the lowest stroke knowledge awareness scores. This profile is characterized by the compounding of multiple barriers: lowest EMS utilization (11.3%), highest living-alone rate (37.7%), high rural residence (45.3%), and the highest inter-hospital transfer rate (41.5%). Patients in this category may face multiple barriers to seeking medical care, such as low health awareness, insufficient family support, and remote residence, leading to severe delays in seeking treatment.

**Table 3:** Comparison of core behavioral indicators for potential profile categories [M (P25, P75)].

Observed variables	Rapid decision-making-emergency transport type (*n* = 69)	Delayed recognition-self-referral TYPE (*n* = 61)	Family involvement-prudent decision-making approach (*n* = 37)	Multiple barriers-delayed access type (*n* = 53)
Time from onset to symptom recognition (minutes)	10.00 (8.00, 13.00)	45.00 (38.50, 54.50)	27.00 (22.50, 32.50)	79.00 (71.25, 90.00)
Time from symptom recognition to decision-making (minutes)	16.00 (11.50, 20.00)	60.00 (53.00, 68.50)	91.00 (84.50, 107.50)	180.00 (161.00, 199.00)
Time from onset to presentation (minutes)	60.00 (50.50, 70.00)	240.00 (211.00, 273.50)	183.00 (171.00, 217.50)	475.00 (437.50, 517.50)
Stroke knowledge awareness score (points)	68.00 (65.00, 70.00)	40.00 (37.00, 43.00)	50.00 (47.00, 52.00)	35.00 (33.00, 37.00)
EMS utilization (*n*, %)	64 (92.75)	15 (24.59)	14 (37.84)	6 (11.32)
Living alone (%)	5 (7.25)	9 (14.75)	5 (13.51)	20 (37.74)
Rural residence (%)	17 (24.64)	29 (47.54)	14 (37.84)	24 (45.28)
Inter-hospital transfer (%)	4 (5.80)	12 (19.67)	13 (35.14)	22 (41.51)

### Comparative demographic characteristics of patients across different potential profiles

Based on seven demographic characteristics from general records, a comprehensive comparison of differences in demographic features across the four profile categories revealed the following (Table 4): gender, occupation, and marital status were evenly distributed across the four profiles, with no statistically significant differences (*p* > 0.05). However, significant differences existed across profiles for age, education level, place of residence, and living alone status (*p* < 0.05). Specifically: age: rapid decision-making-emergency transport profile predominantly comprised middle-aged and young adults, with 45–60 years accounting for 43.48% (30/69) (highest); the multiple barriers-delayed seeking care profile predominantly included elderly individuals, with those aged >75 years accounting for 43.40% (23/53) (highest), showing significant differences (χ^2^ = 14.696, *p* = 0.023); regarding educational attainment, the rapid decision-making-emergency transport profile had a higher proportion of individuals with higher education, with college degree or above reaching 52.17% (36/69) (highest); multiple barriers-delayed seekers showed a high proportion with low education levels, with 39.62% (21/53) at primary school level or below (highest), showing significant differences (χ^2^ = 13.986, *p* = 0.030); regarding residence, rapid decision-making-emergency transporters had 75.36% (52/69) urban residents (highest); Delayed recognition-self-seeking treatment type: rural residents accounted for 47.54% (29/61) (highest), with significant differences (χ^2^ = 8.761, *p* = 0.033); regarding living status, multiple barriers-delayed treatment type: living alone rate was 37.74% (20/53) (highest); while the rapid decision-making-emergency transport group had only 7.25% (5/69) living alone (lowest), with significant differences (χ^2^ = 20.573, *p* < 0.001).

**Table 4:** Comparison of demographic characteristics among patients with different potential profiles [*n* (%)].

Demographic characteristics	Rapid decision-making-emergency transport type (*n* = 69)	Delayed recognition-self-referral type (*n* = 61)	Family involvement-prudent decision-making approach (*n* = 37)	Multiple barriers-delayed access type (*n* = 53)	χ^2^ value	*p* value
Gender					0.736	0.865
M	41 (59.42%)	33 (54.10%)	23 (62.16%)	30 (56.60%)		
F	28 (40.58%)	28 (45.90%)	14 (37.84%)	23 (43.40%)		
Age					14.696	0.023
45–60 year	30 (43.48%)	17 (27.87%)	9 (24.32%)	12 (22.64%)		
61–75 year	28 (40.58%)	26 (42.62%)	18 (48.65%)	18 (33.96%)		
>75 year	11 (15.94%)	18 (29.51%)	10 (27.03%)	23 (43.40%)		
Level of education					13.986	0.030
Elementary school and below	9 (13.04%)	17 (27.87%)	9 (24.32%)	21 (39.62%)		
Junior high school and high school	24 (34.78%)	22 (36.07%)	14 (37.84%)	18 (33.96%)		
Associate degree or higher	36 (52.17%)	22 (36.07%)	14 (37.84%)	14 (26.42%)		
Place of residence					8.761	0.033
City	52 (75.36%)	32 (52.46%)	23 (62.16%)	29 (54.72%)		
Rural	17 (25.64%)	29 (47.54%)	14 (37.84%)	24 (45.28%)		
Living alone					20.573	<0.001
Yes	5 (7.25%)	9 (14.75%)	5 (13.51%)	20 (37.74%)		
No	64 (92.75%)	52 (85.25%)	32 (86.49%)	33 (62.26%)		
Occupation					7.012	0.320
Knowledge workers	22 (31.88%)	13 (21.31%)	7 (18.92%)	9 (16.98%)		
Manual laborer	30 (43.48%)	26 (42.62%)	19 (51.35%)	22 (41.51%)		
Unemployed/Retired	17 (24.64%)	22 (36.07%)	11 (29.73%)	22 (41.51%)		
Marital Status					1.417	0.702
Married with a spouse	60 (86.96%)	52 (85.25%)	31 (83.78%)	42 (79.25%)		
Unmarried/divorced/widowed	9 (13.04%)	9 (14.75%)	6 (16.22%)	11 (20.75%)		

## Comparison of clinical characteristics among patients with different potential profiles

Comprehensive comparison of demographic differences across the four profile categories based on five clinical characteristics from the general data revealed (Table 5): significant differences existed among the four profiles in the distribution of underlying disease count, onset time, and NIHSS scores (*p* < 0.05). Specifically: regarding underlying disease count, the rapid decision-making profile showed the highest proportion (49.28%, 34/69) of patients with 0–1 underlying conditions.

**Table 5:** Comparison of clinical characteristics among patients with different potential profiles [*n* (%)].

Clinical features	Rapid decision-making-emergency transport type (*n* = 69)	Delayed recognition-self-referral type (*n* = 61)	Family involvement-prudent decision-making approach (*n* = 37)	Multiple barriers-delayed access type (*n* = 53)	χ^2^	*p* value
Number of underlying diseases					29.416	<0.001
0–1 species	34 (49.28%)	17 (27.87%)	9 (24.32%)	8 (15.09%)		
2–3 species	26 (37.68%)	28 (45.90%)	21 (56.76%)	20 (37.74%)		
≥4 species	9 (13.04%)	16 (26.23%)	7 (18.92%)	25 (47.17%)		
Primary underlying disease						
Hypertension	46 (66.67%)	47 (77.05%)	27 (72.97%)	41 (77.36%)	2.429	0.488
Hyperlipidemia	30 (43.48%)	29 (47.54%)	17 (45.95%)	28 (52.83%)	1.083	0.781
Diabetes	27 (39.13%)	25 (40.98%)	16 (43.24%)	24 (45.28%)	0.515	0.916
Coronary heart disease	20 (28.99%)	21 (34.43%)	12 (32.43%)	20 (37.74%)	1.096	0.778
Onset time					22.459	0.008
0:00–6:00	4 (5.97%)	6 (9.84%)	3 (8.11%)	17 (32.08%)		
6:00–12:00	27 (40.30%)	20 (32.79%)	12 (32.43%)	12 (22.64%)		
12:00–18:00	21 (31.34%)	16 (26.23%)	12 (32.43%)	11 (20.75%)		
18:00–24:00	17 (25.37%)	19 (31.15%)	10 (27.03%)	13 (24.53%)		
NIHSS score (points)					37.056	<0.001
Mild (0–4 points)	43 (62.32%)	22 (36.07%)	12 (32.43%)	9 (16.98%)		
Moderate (5–15 points)	24 (34.78%)	35 (57.38%)	23 (62.16%)	32 (60.38%)		
Severe (≥16 points)	2 (2.90%)	4 (6.56%)	2 (5.41%)	12 (22.64%)		

The Multiple Impairment profile exhibited the highest prevalence of multiple underlying conditions. The Delayed Presentation profile had the lowest incidence of underlying conditions. Emergency Transport patients had fewer comorbidities, with 49.28% (34/69) having 0–1 comorbidities (highest proportion). Multiple barriers-delayed presentation patients had the highest number of comorbidities, with 47.17% (25/53) having ≥4 comorbidities (highest), with significant differences (χ^2^ = 29.416, *p* < 0.001). Regarding onset time, delayed-presentation patients had the highest proportion of nocturnal onset, with 32.08% (17/53) occurring between 0:00–6:00 (highest), while rapid-decision-emergency transport patients showed the highest incidence during daytime hours, with 40.30% (27/69) occurring between 6:00–12:00 (highest), with significant intergroup differences (χ^2^ = 22.459, *p* = 0.008). Regarding disease severity (NIHSS score), rapid decision-making-emergency transport patients exhibited milder conditions, with mild neurological deficits (0–4 points) accounting for 62.32% (43/69) (highest), multiple impairments-delayed presentation patients had more severe conditions, with severe neurological deficits (≥16 points) accounting for 22.64% (12/53) (highest), with significant differences (χ^2^ = 37.056, *p* < 0.001); Further analysis of primary underlying diseases showed hypertension prevalence ranging from 66.67 to 77.36%, hyperlipidemia from 43.48 to 52.83%, diabetes from 39.13 to 45.28%, and coronary heart disease from 28.99 to 37.74%. The distribution of disease prevalence across groups was balanced, with no statistically significant differences (all *p* > 0.05).

### Comparison of clinical outcomes among patients with different potential profiles

Intergroup comparisons of clinical outcomes (reperfusion therapy rates, length of hospital stay, prognosis) among patients with four distinct presentation profiles revealed significant differences across all three indicators (*p* < 0.001) (Table 6): regarding reperfusion therapy rates, the rapid decision-emergency transport profile exhibited the highest overall reperfusion rate at 63.77% (44/69), comprising 47.83% (33/69) for intravenous thrombolysis and 15.94% (11/69) for mechanical thrombectomy. Mechanical thrombectomy 15.94% (11/69). The multiple barriers-delayed presentation group had the lowest overall reperfusion rate at only 7.55% (4/53), with just 2 cases (3.77%) receiving IV thrombolysis and 2 cases (3.77%) undergoing mechanical thrombectomy. Intergroup differences were significant (χ^2^ = 48.086, *p* < 0.001). Regarding length of hospital stay, the multiple barriers-delayed presentation group had the longest stay at [15 (11, 22)] days, while the rapid decision-emergency transport group had the shortest at [7 (5, 10)] days, with significant intergroup differences (H = 107.600, *p* < 0.001). Regarding prognosis, the Rapid decision-emergency transport group had the highest favorable prognosis rate at 72.46% (50/69), while the multiple barriers-delayed presentation group had the lowest at only 13.21% (7/53), with significant differences between groups (χ^2^ = 44.803, *p* < 0.001).

**Table 6:** Comparison of clinical outcomes among patients with different potential profiles.

Clinical outcome measures	Rapid decision-making-emergency transport type (*n* = 69)	Delayed recognition-self-referral type (*n* = 61)	Family involvement-prudent decision-making approach (*n* = 37)	Multiple barriers-delayed access type (*n* = 53)	H/χ^2^	*p* value
Reperfusion therapy					48.086	<0.001
Intravenous thrombolysis	33 (47.83%)	12 (19.67%)	7 (18.92%)	2 (3.77%)		
Mechanical thrombectomy	11 (15.94%)	3 (4.92%)	3 (8.11%)	2 (3.77%)		
Length of hospital stay (days)	7 (5,10)	10 (7,14)	11 (8,15)	15 (11,22)	107.600	<0.001
Prognosis (mRS score)					44.803	<0.001
Good (0–2 points)	50 (72.46%)	23 (37.70%)	14 (37.84%)	7 (13.21%)		
Poor (3–6 points)	19 (27.54%)	38 (62.30%)	23 (62.16%)	46 (86.79%)		

### Correlation analysis between emergency department presentation patterns and prognosis (mRS score)

To determine the strength of association between core indicators of emergency department presentation patterns and prognosis in AIS patients, this study employed Spearman’s rank correlation analysis. Results (Table 7) demonstrated significant correlations between core presentation indicators and prognosis (mRS score): time from onset to symptom recognition, time from symptom recognition to decision-making, and time from onset to presentation were all significantly positively correlated with mRS scores (r = 0.440, 0.446, 0.458, all *p* < 0.001), whereas stroke knowledge awareness score was significantly negatively correlated with mRS score (r = −0.431, *p* < 0.001).

**Table 7:** Correlation analysis between healthcare-seeking behavior indicators and mRS scores.

Healthcare utilization indicators	Spearman’s correlation coefficient (r)	*p* value	Direction of association with mRS score
Time from onset to symptom recognition (minutes)	0.440	<0.001	Positive correlation (the longer the duration, the higher the mRS score)
Time from symptom recognition to decision-making (minutes)	0.446	<0.001	Positive correlation (the longer the duration, the higher the mRS score)
Time from onset to presentation (minutes)	0.458	<0.001	Positive correlation (the longer the duration, the higher the mRS score)
Stroke knowledge awareness score (points)	−0.431	<0.001	Negative correlation (higher scores correspond to lower mRS scores)

### Multivariate regression analysis of prognosis

To clarify whether latent profile membership independently predicts functional outcome after adjusting for major confounders, multivariate ordinal logistic regression was conducted with discharge mRS as the dependent variable. After adjustment for age, baseline NIHSS score, comorbidity burden, and onset-to-presentation time, latent profile classification remained a significant independent predictor of poor prognosis (higher mRS). Compared with the Rapid Decision-Making–Emergency Transport Profile, the Multiple Barriers–Delayed Presentation Profile was associated with significantly higher odds of worse functional outcome (OR = 4.87, 95% CI: 2.92–8.13, *p* < 0.001). Similar independent adverse associations were observed for the Delayed Recognition–Self-Presentation Profile (OR = 2.15, 95% CI: 1.30–3.55, *p* = 0.003) and the Family Involvement–Cautious Decision-Making Profile (OR = 1.88, 95% CI: 1.07–3.30, *p* = 0.027). Among covariates, older age, higher baseline NIHSS score, greater comorbidity burden, and longer onset-to-presentation time were also independently associated with worse mRS (all *p* < 0.05). These results confirm that latent profile classification provides incremental prognostic value beyond established clinical predictors (Table 8).

**Table 8:** Multivariate ordinal logistic regression for discharge mRS.

Variables	OR (95% CI)	*p* value
Latent profile (ref: rapid decision-making–emergency transport)		
Delayed recognition–self-presentation	2.15 (1.30–3.55)	0.003
Family involvement–cautious decision-making	1.88 (1.07–3.30)	0.027
Multiple barriers–delayed presentation	4.87 (2.92–8.13)	<0.001
Age (per 10-year increase)	1.32 (1.11–1.57)	0.002
Baseline NIHSS (per 1-point increase)	1.18 (1.10–1.27)	<0.001
Comorbidity burden (≥4 vs. 0–1)	1.94 (1.21–3.11)	0.006
Onset-to-presentation time (per 60 min increase)	1.26 (1.14–1.39)	<0.001

## Discussion

This study employed LPA to systematically identify four latent profiles of emergency department presentations among AIS patients for the first time. It revealed the association patterns between presentation heterogeneity and demographic, clinical characteristics, and prognosis, providing critical evidence for optimizing AIS emergency care pathways and developing stratified intervention strategies.

The profile classification results show that the four profiles—“Rapid decision-making-emergency transport type,” “Delayed recognition-self-presentation type,” “Family involvement-cautious decision-making type,” and “Multiple barriers-delayed presentation type”—accurately cover the behavioral differences across the entire process from “symptom recognition-decision to seek care to transport and admission.” now augmented by critical information on EMS use, living situation, geographic access, and transfer patterns. The distribution of these categories reflects clinical reality: nearly half of patients experience significant delays in seeking care, aligning closely with the overall delay in AIS patient treatment in China (16, 17).

Based on the core characteristics of each profile, patients classified as “Rapid decision-making-emergency transport type” (31.36%) demonstrated an efficient action chain of “short-time recognition-rapid decision-making-emergency transport” efficient behavioral chain. Their median time from onset to hospital arrival was only 60 min, with a stroke knowledge awareness score of 68 (out of 75). EMS utilization reached 92.75%, and direct presentation to our hospital was 94.20%, contributing to the shortest onset-to-presentation times. Both the reperfusion therapy rate (63.77%) and favorable prognosis rate (72.46%) were the highest among the four categories. This outcome validates the positive feedback loop of “knowledge-awareness-action”: high knowledge levels enabled patients and families to rapidly recognize typical AIS symptoms (e.g., hemiplegia, speech impairment), while choosing emergency transport further shortened transit time, perfectly aligning with the “time is brain” treatment principle (18, 19). Demographically, this group predominantly comprised middle-aged and young adults (45–60 years old: 43.48%), highly educated individuals (college degree or higher: 52.17%), urban residence (75.36%), and non-single living arrangements (92.75%). This suggests that health awareness among younger populations, information access facilitated by higher education, and more readily available emergency resources in urban areas collectively form the conditions supporting efficient medical care. This provides a clear direction for targeting subsequent intervention strategies (20, 21).

Patients with delayed recognition who self-presented (27.73%) primarily faced issues of “delayed symptom recognition + inappropriate transport methods”: The time from onset to symptom recognition reached 45 min, with 60 min elapsing from symptom recognition to decision-making. Most patients opted for self-transport (non-emergency transfer), ultimately resulting in a total time from onset to presentation of 240 min. This significantly exceeded the optimal time windows for intravenous thrombolysis (4.5 h) and mechanical thrombectomy (6–24 h). Their stroke knowledge score was only 40 points, indicating insufficient disease awareness as the root cause-patients may misinterpret AIS symptoms as “transient discomfort” (e.g., fatigue, dizziness), delaying decision-making (22, 23); The choice of “self-presentation” further exposed a lack of emergency awareness. Studies indicate that self-presentation may prolong overall treatment time due to inadequate professional care and coordination (24, 25). Geographically, rural areas accounted for 47.54% of these patients (the highest among the four categories), reflecting inadequate emergency knowledge dissemination and low accessibility of emergency resources in rural regions. Targeted health education initiatives for rural populations are urgently needed (26, 27).

Patients classified as “Family involvement-cautious decision-making type” (16.82%) were characterized by the longest decision-making time (median 91 min). Despite moderate symptom recognition time (27 min) and knowledge level (50 points), excessive caution among family members during decision-making led to delayed medical care. Notably, this profile had the highest inter-hospital transfer rate (35.14%), suggesting that initial presentation to non-stroke centers-possibly due to family decisions to go to the nearest hospital rather than a stroke-ready center-exacerbates delays. This phenomenon may relate to two factors: First, family members hesitated in judging the necessity of seeking medical care, with some preferring to “observe whether symptoms resolve” rather than seek immediate treatment. Second, “disagreements” within family decision-making delayed the optimal treatment window (28, 29). Furthermore, most patients in this group had 2–3 underlying conditions (56.76%), suggesting family members may have concerns about “stroke treatment risks” due to multiple chronic comorbidities, further intensifying decision hesitation (30, 31). This profile highlights for the first time the critical role of “family decision-making” in AIS care, breaking the limitation of previous studies that focused solely on the patient.

Patients with “multiple barriers-delayed presentation” (24.09%) exhibited the poorest prognosis: the time from symptom onset to hospital arrival reached 475 min (nearly 8 h), stroke knowledge awareness scored only 35 points, reperfusion therapy rate was merely 7.55%, and the rate of poor outcomes soared to 86.79%. Their predicament stems from the compounding effects of “physiological-cognitive-social” barriers: Physiologically, these patients predominantly featured advanced age (>75 years old: 43.40%), multiple comorbidities (≥4 conditions: 47.17%), and severe disease (high NIHSS scores: 22.64%). They exhibited poor symptom awareness, with comorbidities potentially masking AIS symptoms. Cognitively, low educational attainment (39.62% with primary school or below) leads to insufficient disease awareness and difficulty recognizing symptoms. Socially, a high rate of living alone (37.74%, highest among the four categories) results in a lack of timely detection and assistance from family members. Structurally, low EMS utilization (11.32%, lowest), high rural residence (45.28%), and the highest inter-hospital transfer rate (41.51%) indicate that geographic distance and fragmented care pathways compound individual-level delays. Furthermore, 32.08% of cases occur at night (highest among the four categories), further reducing the probability of being discovered. This profile indicates that delayed AIS presentation stems not from a single factor but from multidimensional deficits in “individual capacity-family support-social resources,” necessitating the development of a systematic intervention framework (32).

This study employed Spearman correlation analysis to establish quantitative associations between treatment-seeking indicators and prognosis (mRS score): time from onset to presentation, time from onset to symptom recognition, and time from symptom recognition to decision-making showed significant positive correlations with mRS score (r = 0.440, 0.446, 0.458, all *p* < 0.001). while stroke knowledge awareness scores showed a significant negative correlation with mRS scores (r = −0.431, *p* < 0.001). Critically, multivariate regression confirmed that latent profile membership remained an independent predictor of functional outcome after adjustment for age, baseline NIHSS, comorbidity burden, and onset-to-presentation time. The Multiple Barriers–Delayed Presentation Profile was associated with nearly fivefold higher odds of poor prognosis compared with the Rapid Decision-Making–Emergency Transport Profile, demonstrating that latent classification provides incremental prognostic value beyond traditional clinical variables. This finding provides direct evidence for the causal chain linking “time-knowledge-outcome.” From a pathophysiological perspective, the core pathology of AIS is cerebral tissue ischemia and hypoxia. Once ischemia exceeds a critical threshold (e.g., irreversible damage within the core area of cerebral infarction within 6 h), even if blood flow is restored, it is difficult to salvage impaired neurological function (33). Patients classified as “rapid decision-making-emergency transport” received timely care, enabling more to undergo reperfusion therapy within the time window. This effectively reduced infarct size and improved neurological function. In contrast, “multiple barriers-delayed presentation” patients experienced delayed care, with most missing the reperfusion window and receiving only symptomatic supportive treatment, resulting in poorer outcomes (34–36). Regarding the mechanism of knowledge impact, patients with high stroke knowledge scores not only rapidly recognize symptoms but also accurately judge the need for “immediate medical attention,” opting for emergency transport. This creates a positive cycle: “knowledge → correct behavior → timely treatment → favorable prognosis.” Conversely, patients with insufficient knowledge may experience delays even with typical symptoms due to “unrecognized stroke” or “unaware of the need for emergency care, “ultimately leading to poor outcomes. This finding aligns with previous research, further confirming that health knowledge serves as an” upstream intervention target” for improving AIS prognosis (37, 38).

Based on core barriers across four patient profiles, targeted intervention strategies are proposed: Rapid decision-making-emergency transport type: focus on “secondary prevention” by reducing recurrence risk through regular follow-ups and medication reminder apps; delayed recognition-self-seeking care type: Implement “symptom recognition + emergency subsidy” interventions, such as creating “illustrated atypical symptom manuals” and providing ambulance fee subsidies for rural patients, aiming to shorten symptom recognition time; Family involvement-cautious decision-making type: Establish a “family decision support system” by launching emergency hotlines and providing regional thrombolysis hospital maps to reduce decision hesitation; Multiple barriers-delayed presentation type: Implement “end-to-end support” by equipping elderly patients living alone with “smart alert bracelets” (automatically calling emergency services during abnormalities) and establishing “stroke emergency points” in rural areas to shorten transport times. This study also has certain limitations: First, although multivariate regression adjusted for key confounders (age, NIHSS, comorbidity burden, and onset-to-presentation time), residual confounding from unmeasured variables (e.g., socioeconomic status, preadmission disability) cannot be fully excluded. Second, this is a single-centre study with convenience sampling, which may lead to selection bias and limit the representativeness of the sample. Therefore, the findings of this study should be considered hypothetical rather than generalisable to broader populations. Third, Third, the primary outcome was assessed using discharge mRS instead of the standard 90-day mRS, which is the recommended primary endpoint for stroke studies. The lack of long-term functional follow-up data significantly restricts the interpretation of sustained functional outcomes and limits the generalizability of prognostic conclusions. While this study expanded the model to include EMS utilization, living status, residential area, and transfer pathway, other potential influencing factors such as economic status and exact distance to emergency resources were not included. Fourth, which may have omitted some key variables. Future research will need to conduct multi-center, large-scale sample studies to verify the universality of this characteristic description, and conduct long-term follow-ups to observe the differences in long-term outcomes among patients with different characteristics as well as the sustained effects of intervention measures.

In summary, this study employs LPA to identify four distinct heterogeneous profiles among AIS patients presenting to emergency departments for the first time, using eight-indicator model that captures temporal, cognitive, behavioral, and structural dimensions of prehospital care. It reveals significant differences in demographic, clinical characteristics, and outcomes across these profiles, while confirming the strong association between presentation time, stroke knowledge, and prognosis. Crucially, the inclusion of EMS utilization, living status, geographic accessibility, and transfer pathways demonstrates that classification based solely on temporal delays is insufficient; multidimensional indicators yield greater clinical interpretability and actionable insights. The findings suggest that emergency management of AIS requires moving beyond a “one-size-fits-all” approach. Instead, stratified intervention strategies should be developed based on patient profiles, with a particular focus on the needs of rural, low-educated, elderly, and solitary living patients. A multidimensional intervention encompassing “knowledge education-optimization of emergency resources-reinforcement of family support” can reduce presentation delays, increase reperfusion therapy rates, and ultimately improve the overall prognosis of AIS patients. Future multi-center, prospective studies are needed to validate these patient profiles and intervention outcomes, providing stronger evidence for establishing a “precision management system” for emergency care of AIS in China.

## Data Availability

The original contributions presented in the study are included in the article/supplementary material, further inquiries can be directed to the corresponding author.
